# Oxidative stress and biomarker of TNF-α, MDA and FRAP in hypertension

**DOI:** 10.25122/jml-2019-0031

**Published:** 2019

**Authors:** Manish Kumar Verma, Anoop Jaiswal, Preeti Sharma, Pradeep Kumar, Anand Narayan Singh

**Affiliations:** 1. Department of Biochemistry, G.S.V.M. Medical College Kanpur, India; 2. Department of Biochemistry, Moti Lal Nehru Medical College, Allahabad, India; 3. Department of Biochemistry, Santosh Medical College and Hospital (Santosh University), Ghaziabad, India

**Keywords:** hypertension, blood pressure, reactive oxygen species, biomarker, NO – Nitric oxide, ML – Milliliter, TNF-α – Tumor Necrosis Factor, MDA – Malondialdehyde, FRAP – Ferric reducing antioxidant power, ROS – Reactive Oxygen Species

## Abstract

Concurrent with the misbalance of oxidizing agents and antioxidants, high blood pressure is a major physical burden condition in the current scenario. Tumor necrosis factor-α (TNF-α) plays a vital role in the pathogenesis of hypertension. Tumor necrosis factor-α, inhibitor improves clinical symptoms however their outcome on high blood pressure has not been investigated. We investigated the inflammatory marker TNF-α, malondialdehyde (MDA) and ferric reducing antioxidant power (FRAP) in hypertensive patients. We measured randomly blood pressure using an ambulatory observe in hypertensive patients, measured systolic BP X 140 mmHg and/or diastolic BP X 90 mmHg were considered hypertensive. Total 60 cases were considered in the study that involves 30 hypertensive patients and 30 normal control. Measurements of serum concentrations of TNF-α, MDA, FRAP in hypertension patients was done in both the groups. Serum TNF-α was found to be remarkably increased in study subjects as compared to normal group (r=0.32, p<0.0001*). Serum MDA was also raised in hypertensive as compared to control (r=0.99**, p<0.0001*). While Serum FRAP was found to be decreased in hypertensive group in comparison to healthy control (r=0.23, p<0.0001*). It is concluded that high blood pressure leads to generation of oxidative stress with remarkable elevation of TNF-α and malondialdehyde levels. While reduced FRAP indicates its probable role in lipid peroxidation and in the pathogenesis of hypertension.

## Introduction

Hypertension is a main health problem in developed as well as developing countries with a common end result of elevated blood pressure (BP). Hypertension (HTN) is present in 60–70% of the population over 60 years of age and may result in cardiovascular complications such as stroke, coronary heart disease, and heart failure.

High blood pressure (essential hypertension) is defined as systolic pressure >140 and/or diastolic pressure >90. Patients with systolic blood pressure (SBP) between 120 and 139, or diastolic blood pressure (DBP) of 80–89 are considered “pre-hypertensive” and need medical monitoring and lifestyle changes [1]. Oxidative stress is the state of imbalance between the ROS and the ability of the biological system to detoxify readily the reactive intermediates that improves vascular oxidative stress which could be consider to the pathogenesis of high blood pressure – a major jeopardy factor for heart disease mortality [2, 3].

Oxidative stress occurs while an imbalance between the generation of ROS, the antioxidant protection systems so that the latter become overwhelmed [4, 5]. In human, high blood pressure ROS may raise due to a decrease in the activity of antioxidant enzymes [6]. The significance of reactive oxygen in the development of high blood pressure have been recently reviewed [7, 8]. The relationship between high blood pressure, oxidative stress and antioxidants is complex and inadequately understood. Oxidative stress may play a role in the pathophysiology of hypertension. Human and animal studies have demonstrated that HT is accompanied by increase in oxidative stress.

However, the evidence for the above in humans is not clearly define [9]. Studies demonstrate that hypertension may develop as a result of increased reactive oxygen species [10] and that a variety of antioxidant therapies ameliorate hypertension. Hypertensive effects of oxidative stress are mostly due to endothelial dysfunction resulting from disturbances of vasodilator systems, particularly degradation of NO by oxygen-free radicals [11, 12].

Other studies aim to measure the levels of MDA during hypertensive conditions. Elevated serum MDA levels were found in hypertensive patients as compared to normotensive control individuals [13]. Elevated levels of serum MDA and decreased catalase activity were found in hypertensive pregnant women as compared to healthy person [14]. El-Banaet et al. [15] studied the maternal and cord plasma concentration of MDA in pre-clamptic and healthy pregnant women.

The concentration of MDA in pre-clamptics was found to be significantly lower in cord plasma as compared to maternal plasma (the fetus from oxidative injury due to increased oxidative stress of a pre-clamptic mother). MDA is a useful biomarker for lipid peroxidation and oxidative stress. Increased levels of oxidative stress have been associated with various disease patterns.

Ferric reducing antioxidant power had the highest correlations with blood pressure among the oxidative stress-related parameters studied, because of the relationship between oxidative stress and hypertension; it is worth noting that drugs with antioxidant effects can also be expected to lower blood pressure [16]. In addition, the administration has been shown to cause a decrease in oxidative stress in hypertensive [17, 18]. Along these lines, antioxidant vitamins have been shown to exert antihypertensive effects in spontaneous hypertension, although the extensibility of these results to human beings remain controversial [19].

Despite the progress in its diagnosis and treatment, the etiology of HT remains unclear and a matter of substantial debate. It is widely acknowledged that function of the vascular system, kidneys and sympathetic nervous system is critical for control and maintenance of BP [20]. Vascular resistance, stiffness and remodeling as well as endothelial dysfunction are hallmarks of HT [21–24]. Our study was to elucidate the MDA and FRAP in hypertension with and without hypertension.

## Materials and Methods

Clinically diagnosed and confirmed cases of hypertension in the age group of 25–74 years. The study was approved by the institute ethics committee, and informed consent was obtained from all the cases and controls.

**Inclusion:** In this group which includes hypertensive patients with average blood pressure ≥140/90 mmHg for a period >10 years, as defined by JNC 7th criteria [25].

**Exclusion:** Patients with renal disorders, diabetes mellitus, liver disorder, gout, familial hyperlipidemia were expelled. Also patients those who were on antioxidants, vasoactive medicine, lipid lowering statins were expelled.

**Sample collection and storage:** Under aseptic conditions 5 ml of whole blood were collected. Out of this 1 ml was collected in fluoride vial to estimate blood sugar fasting and 4 ml collected without anticoagulant (plain) to estimate MDA, FRAP, TNF-α. To estimate serum creatinine and blood sugar fasting blood is centrifuged (3,000 rpm, for 3–5 min at 37 °C) to obtain serum that was also stored at -80 °C for further biochemical measurements**.**

**Numbers of cases selected for the study were**

• 30 cases of hypertension patients
• 30 controls of normal persons

**Biochemical measurement**

1. Estimation of malondialdehyde by Satoh K (1978) method [26].
2. Ferric reducing antioxidant power (FRAP) assay kit [27].
3. Estimation of human TNF-α immunoassay by sandwich enzyme immunoassay [28].
4. Blood pressure measured by sphygmomanometer.

## Results

Total 60 samples were considered in the study out of which 30 were hypertension patients and 30 were normal individuals. In hypertension group, 11 (36.7%) patients were female and 19 (36.3%) were male whereas in normal group, there were 12 (40%) male and 18 (40.0%) were female persons (Table 1).

**Table 1:** Frequency distribution among male and female in normotensive persons

Gender	Frequency (%)	Valid Percent
Hypertension Patients (n=30)		
Female	11(36.7)	36.7
Male	19(63.3)	63.3
Total	30(100.0)	100.0
Normotensive Patients (n=30)		
Female	12(40)	40
Male	19(60)	60
Total	30(100.0)	100.0

Chi square test

In normal individual group, all the serum values were in normal range. The MDA levels of hypertension patients and normotensive (control) group was extremely significance (p<0.0001). The FRAP levels of hypertension patients and normotensive (control) group was highly significance (p<0.0001). The TNF-α levels of hypertension patients and normotensive group was highly significance (p<0.0001). The SBP levels of hypertension patients and normotensive (control) group was highly significance (p<0.0001). The DBP levels of hypertension patients and normotensive (control) group was highly significance (p<0.0001) (Table 2).

**Table 2:** Clinical characteristics of normotensive subjects and hypertensive patients participating in the study.

Parameter	Hypertension mean±SD (n=30)	Normotensive mean±SD (n=30)	p-Value	t-Value
Age	54.73±11.8	51.23±13.2	0.2834	1.0827
MDA	4.77±0.47	1.89±0.48	0.0001**	23.4812
FRAP	314.7±4.18	432.34±4.83	0.0001**	100.8739
TNF-α	5.01±1.11	2.12±1.35	0.0001**	9.0569
FBS	104.87±7.53	98.33±8.35	0.0023*	3.1858
SBP	161.51±8.57	122.01±6.55	0.0001**	20.0881
DBP	100.53±8.42	84.11±3.10	0.0001**	12.4653

Unpaired t-test, **Statistically highly significant, *Significant.

Table 3 shows the correlation matrix which represents the quantitative measurements of degree of relationship among different variables. It showed that MDA and FRAP were mildly correlated (r = 0.118, P = 0.536) in cases. There is a negative correlation between MDA and SBP (r = −0.073, P = 0.0700). There is a negative correlation between FRAP and TNF-α (r = −0.040, P = 0.834). There is a negative correlation between FRAP and SBP (r = −0.173, P = 0.360). There is a negative correlation between FBS and TNF-α (r = −0.194, P = 0.303). There is a negative correlation between SBP and FRAP (r = −0.173, P = 0.360). There is a negative correlation between SBP and TNF-α (r = −0.092, P = 0.629).

**Table 3:** Pearson correlation coefficient among the biochemical parameters in cases

		FRAP (μmol/l)	TNF-α (ng/mL)	FBS (mg/dl)	SBP (mmHg)	DBP (mmHg)
MDA (µmoll)	Pearson correlation	0.118	0.164	0.139	–0.073	0.148
	Sig. (2-tailed)	0.536	0.385	0.465	0.700	0.434
	n	30	30	30	30	30
FRAP (μmol/l)	Pearson correlation		–0.040	0.048	–0.173	0.189
	Sig. (2-tailed)		0.834	0.801	0.360	0.318
	n		30	30	30	30
TNF-α (ng/mL)	Pearson correlation			–0.194	–0.092	0.020
	Sig. (2-tailed)			0.303	0.629	0.917
	n			30	30	30
FBS (mg/dl)	Pearson correlation				-0.075	–0.150
	Sig. (2-tailed)				0.695	0.428
	n				30	30
SBP (mmHg)	Pearson correlation					0.013
	Sig. (2-tailed)					0.944
	n					30

Table 4: shows the correlation matrix which represents the quantitative measurements of degree of relationship among different variables. It showed that MDA and FRAP were mildly correlated (r = 0.329, P = 0.076) in control group. There is a negative correlation between MDA and TNF-α (r = −0.443, P = 0.014). There is a negative correlation between FBS and TNF-α (r = −0.111, P = 0.559). There is a negative correlation between MDA and DBP (r = −0.038, P = 0.842). There is a negative correlation between FRAP and TNF-α (r = −0.348, P = 0.060). There is a negative correlation between DBP and FRAP (r = −0.254, P = 0.176). There is a negative correlation between TNF-α to FBS, SBP, DBP (r = −0.142, P = 0.453; r= -0.161, P = 0.0.396 r= -0.012 P = 0.948).

**Table 4:** Pearson correlation coefficient among the parameters in controls

		FRAP (μmol/l)	TNF-α (ng/mL)	FBS (mg/dl)	SBP (mmHg)	DBP (mmHg)
MDA (µmol/l)	Pearson correlation	0.329	–0.443	–0.111	0.331	–0.038
	Sig. (2-tailed)	0.076	0.014	0.559	0.074	0.842
	n	30	30	30	30	30
FRAP (μmol/l)	Pearson correlation		–0.348	0.184	0.204	–0.254
	Sig. (2-tailed)		0.060	0.331	0.279	0.176
	n		30	30	30	30
TNF-α (ng/ml)	Pearson correlation			–0.142	–0.161	–0.012
	Sig. (2-tailed)			0.453	0.396	0.948
	n			30	30	30
FBS (mg/dl)	Pearson correlation				0.070	0.286
	Sig. (2-tailed)				0.713	0.126
	n				30	30
SBP (mmHg)	Pearson correlation					0.195
	Sig. (2-tailed)					0.302
	n					30

## Discussion

The primary study is done to reveal that the coronary endothelial dysfunction is separately associated with elevation of the plasma proinflammatory cytokines TNF-α in patients with hypertension. In addition, elevated plasma TNF-α levels strength be helpful to identify the elevated risk of high blood pressure patients with coronary endothelial dysfunction.

In their study, we have determined the levels of some endogenous antioxidants is important for hypertensive subjects when compared with their age and sex matched healthy persons. Our study is the first to evaluate the correlation among necrosis factor and oxidative stress markers in hypertensive patients. Oxidative stress is an occurrence which is moreover due to excessive production of reactive oxygen species. Undernourishment can lead to reduction of antioxidant [29–32].

Oxidative stress is caused by imbalance between the production of reactive oxygen species (ROS) and the ability of a biological system to readily detoxify the reactive intermediates improved vascular oxidative stress, the resulting damage. This could be considered to the pathogenesis of high Blood Pressure, a major factor cause for heart disease mortality. [33] . Because of the relationship between oxidative stress and high blood pressure, it is worth noting that drugs with antioxidant effects can also be expected to lower blood pressure.

Accordingly, the antihypertensive effects of statins could arise from their antioxidant properties, through their ability to reduce the expression of Nicotinamide adenine dinucleotide phosphate (NADPH) oxidase subunits and up regulate catalase expression *in vivo*. Along these lines, antioxidant vitamins have been shown to exert antihypertensive effects in spontaneously hypertensive rats, although the extensibility of these results to human beings remain controversial, and awaits the completion of large scale clinical trials that are currently underway.

The present study justified that FRAP was not elevated in hypertensive patients compared with healthy controls and that there was negative association between TNF-α (r=-0.040 p=0.834) and coronary endothelial dysfunction. This observation indicates that TNF-α, rather than FRAP and MDA, are useful for identifying coronary vascular dysfunction in hypertensive patients.

**Figure 1:**
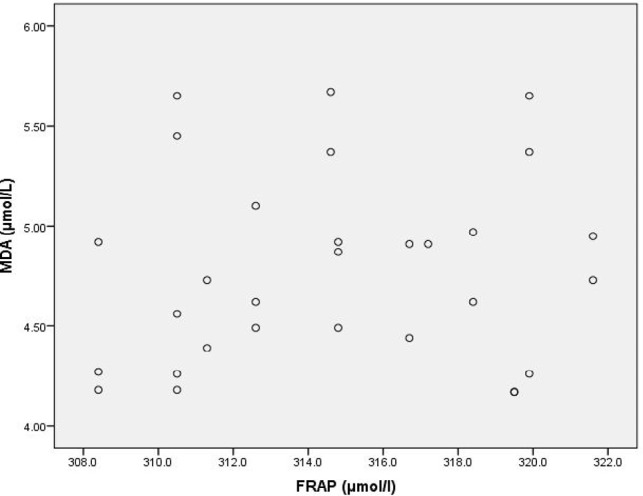
Scatter Diagram Showing Association between MDA and FRAP in Cases

**Figure 2:**
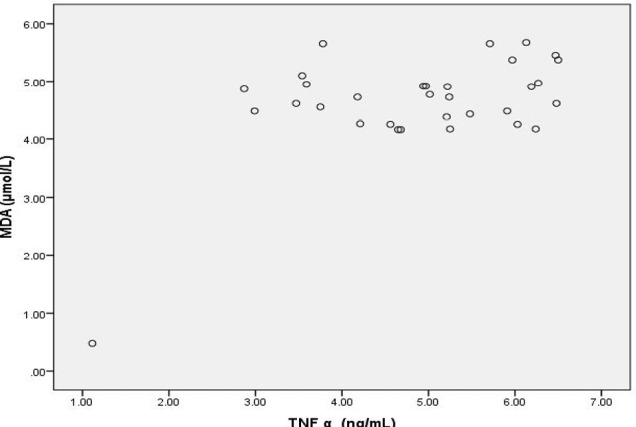
Scatter Diagram Showing Association between MDA and TNF α in cases

**Figure 3:**
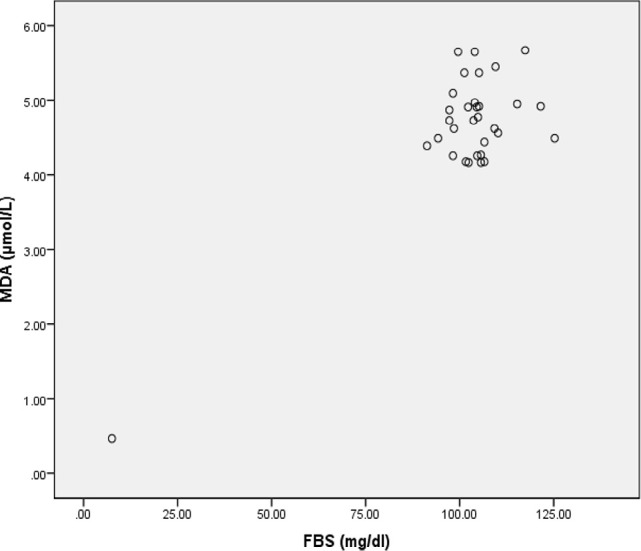
Scatter Diagram Showing Association between MDA and FBS in Cases

**Figure 4:**
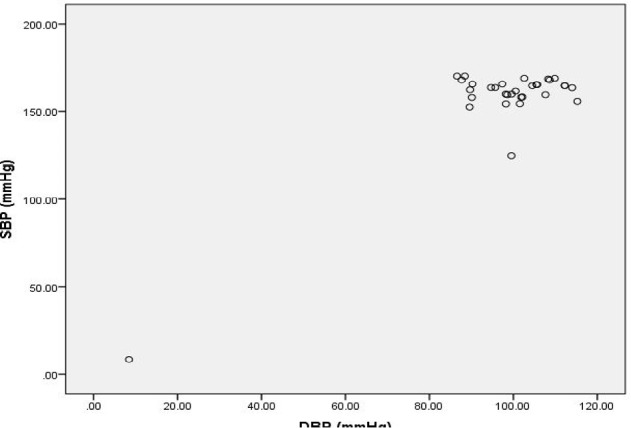
Scatter diagram showing association between SBP and DBP in Cases

**Figure 5:**
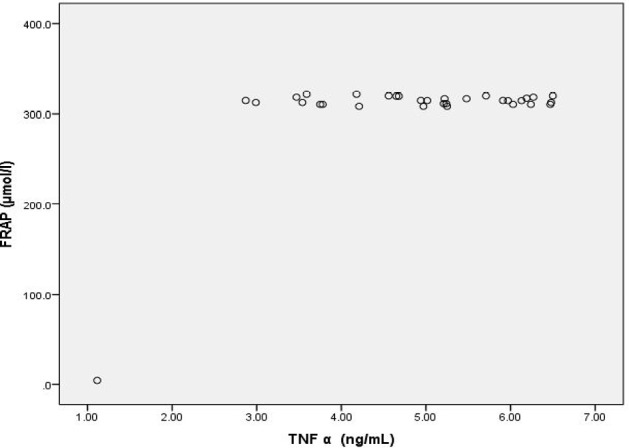
Scatter Diagram Showing Association Between FRAP and TNF-α in Cases

**Figure 6:**
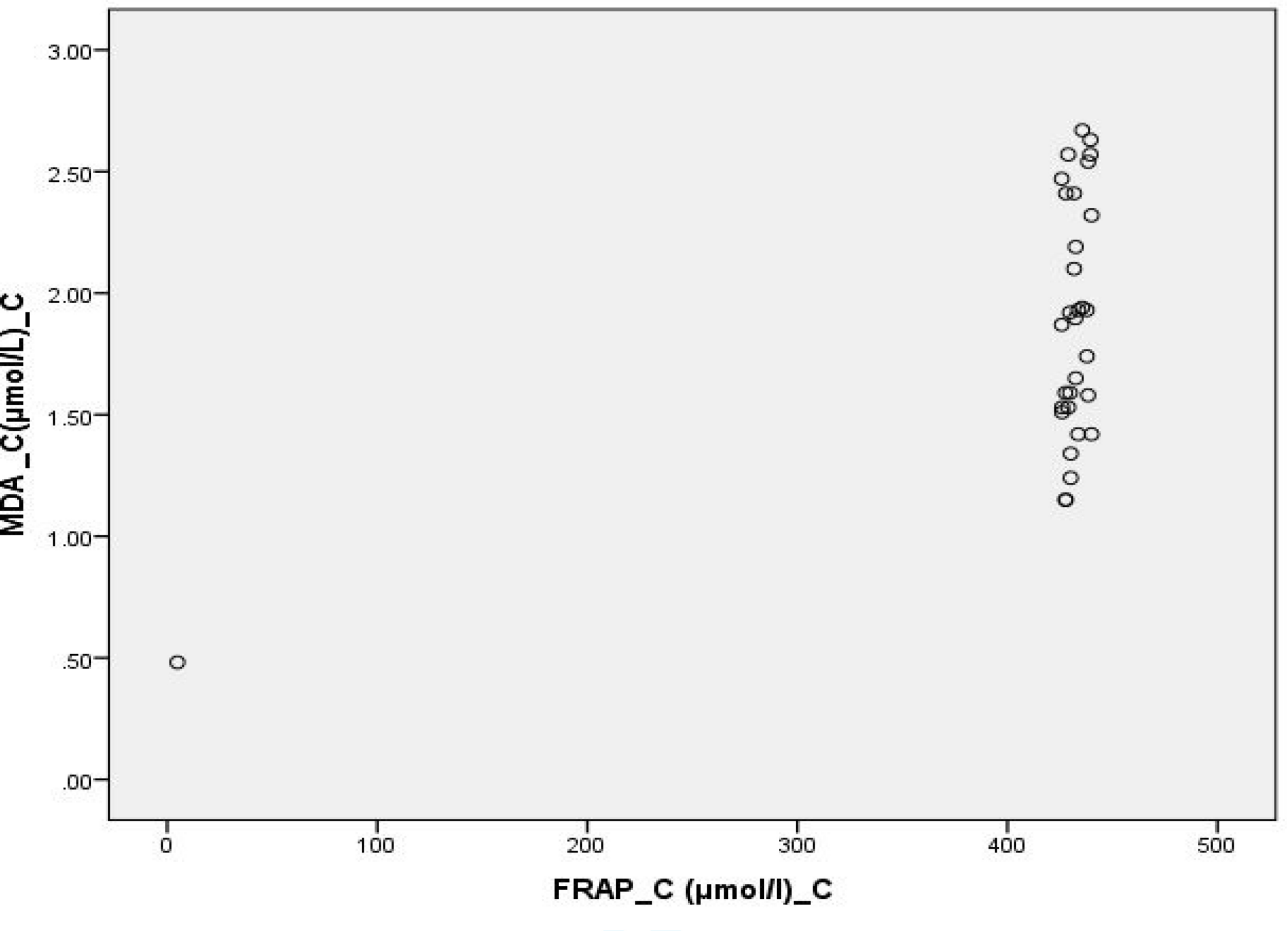
Scatter Diagram Showing Association between MDA and FRAP in Controls

**Figure 7:**
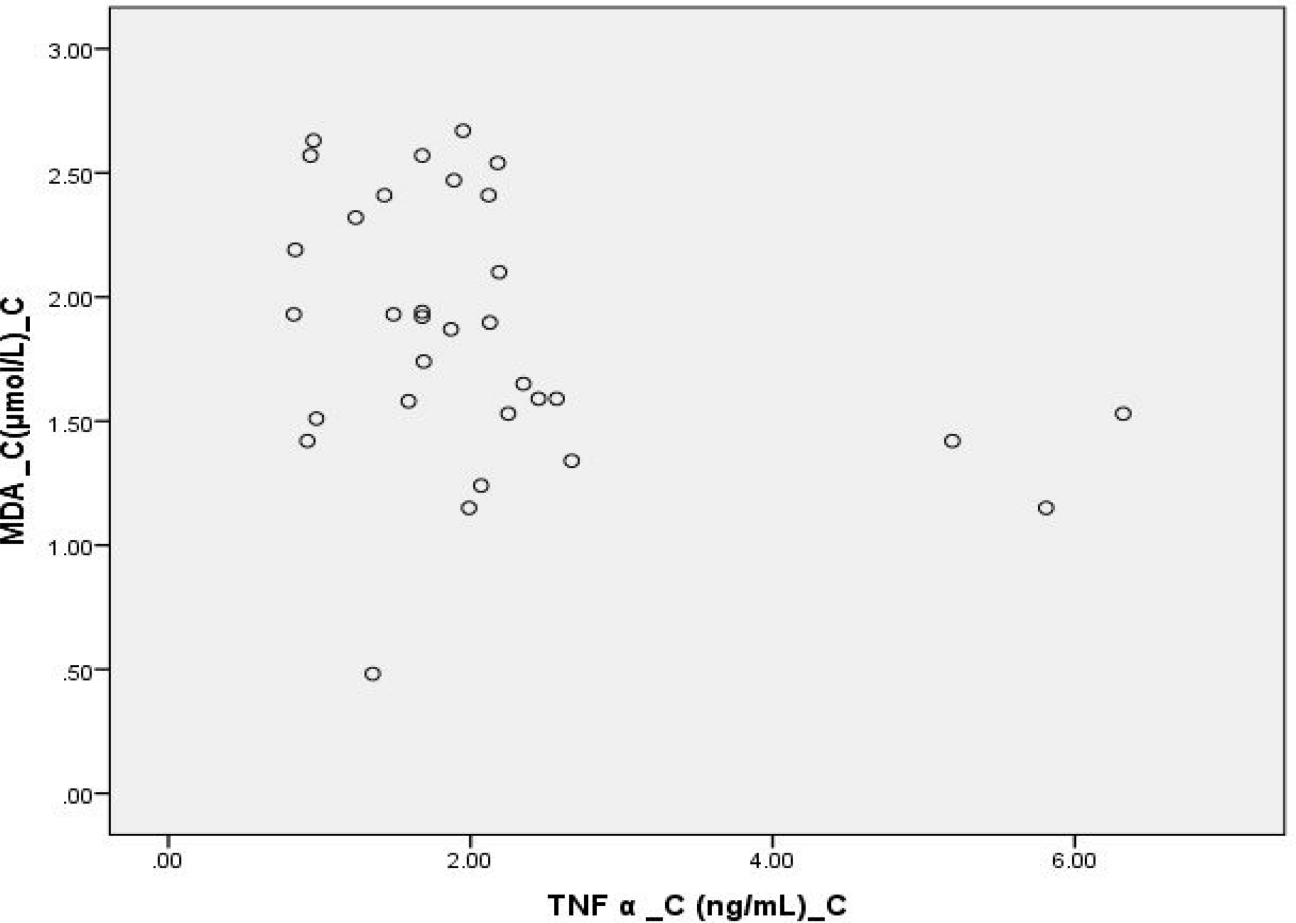
Scatter Diagram Showing Association between MDA and TNF α In Controls

**Figure 8:**
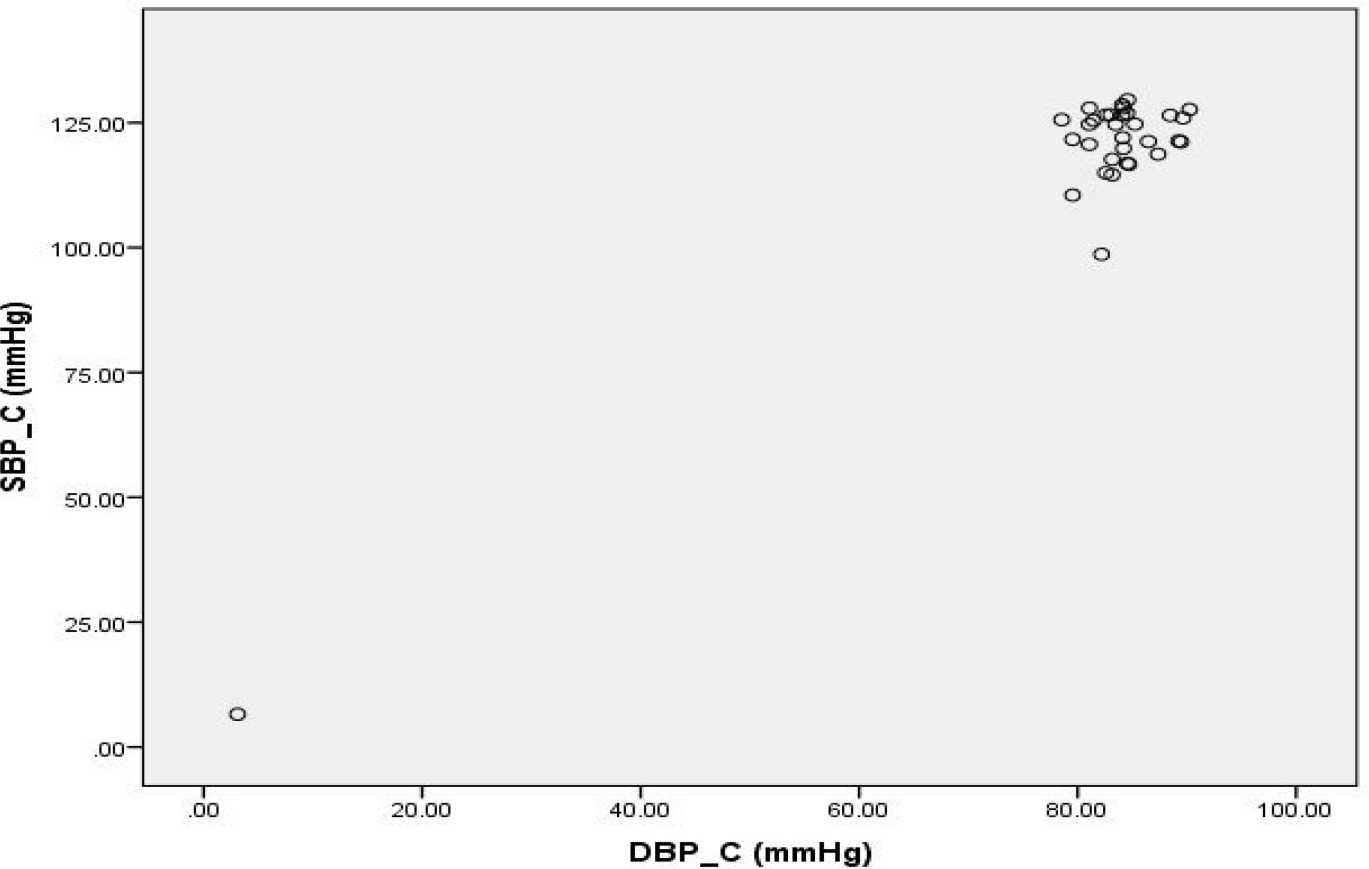
Scatter Diagram Showing Association between SBP and DBP in Controls

**Figure 9:**
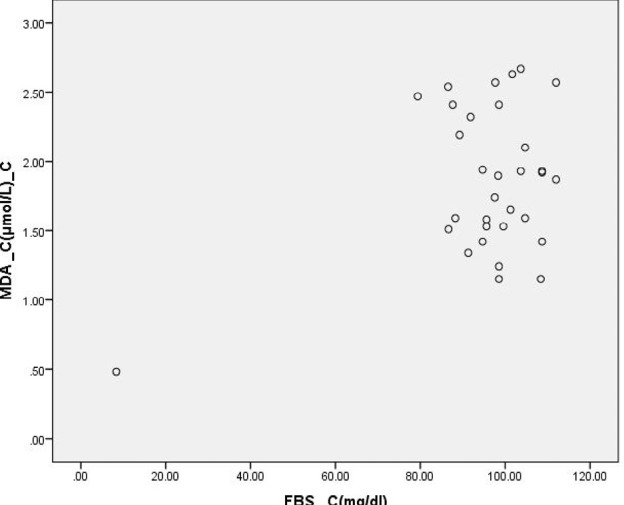
Scatter Diagram Showing Association between MDA and FBS in Controls

## Conclusions

Our studies showed that the present study was reported serum TNF- levels are increase prominent in HTN, and MDA significantly elevated and FRAP was decrease. In addition, it is supplementary to the recent literature in hold of oxidative stress having a pathogenic role in the growth of Hypertension and recommends antioxidants therapies and antioxidants intake of diet. The pathophysiological substrate of these interrelationships needs further investigation through large scale prospective studies.

## Acknowledgement

All the authors duly acknowledge the support of management for designing and writing of the manuscript.

## Financial Disclosure

None

## Contribution of Authors

Review concept – P K & MKV

Review design – ANS, MKV & PS

Supervision – PK & ANS

Materials – AJ

Literature search – MKV, PK & ANS

Writing article – MKV

Critical review – PS & ANS

Article editing – MKV, PS & ANS

Final approval – PK, ANS & PS

## Conflict of Interest

The authors confirm that there are no conflicts of interest.
